# Expectations, perceptions, and physiotherapy predict prolonged sick leave in subacute low back pain

**DOI:** 10.1186/1471-2474-10-139

**Published:** 2009-11-13

**Authors:** Silje E Reme, Eli M Hagen, Hege R Eriksen

**Affiliations:** 1 Research Center for Health Promotion, Faculty of Psychology, University of Bergen, Norway; 2 Unifob Health, University Research Bergen, Norway; 3 Department of Psychiatry, Haukeland University Hospital, Bergen, Norway; 4 Spine Clinic, Sykehuset Innlandet HF, Ottestad, Norway

## Abstract

**Background:**

Brief intervention programs for subacute low back pain (LBP) result in significant reduction of sick leave compared to treatment as usual. Although effective, a substantial proportion of the patients do not return to work. This study investigates predictors of return to work in LBP patients participating in a randomized controlled trial comparing a brief intervention program (BI) with BI and physical exercise.

**Methods:**

Predictors for not returning to work was examined in 246 patients sick listed 8-12 weeks for low back pain. The patients had participated in a randomized controlled trial, with BI (n = 122) and BI + physical exercise (n = 124). There were no significant differences between the two intervention groups on return to work. The groups were therefore merged in the analyses of predictors. Multiple logistic regression analysis was used to identify predictors for non return to work at 3, 12, and 24 months of follow-up.

**Results:**

At 3 months of follow-up, the strongest predictors for not returning to work were pain intensity while resting (OR = 5.6; CI = 1.7-19), the perception of constant back strain when working (OR = 4.1; CI = 1.5-12), negative expectations for return to work (OR = 4.2; CI = 1.7-10), and having been to a physiotherapist prior to participation in the trial (OR = 3.3; CI = 1.3-8.3). At 12 months, perceived reduced ability to walk far due to the complaints (OR = 2.6; CI = 1.3-5.4), pain during activities (OR = 2.4; CI = 1.1-5.1), and having been to a physiotherapist prior to participation in the trial (OR = 2.1; CI = 1.1-4.3) were the strongest predictors for non return to work. At 24 months age below 41 years (OR = 2.9; CI = 1.4-6.0) was the only significant predictor for non return to work.

**Conclusion:**

It appears that return to work is highly dependant on individual and cognitive factors. Patients not returning to work after the interventions were characterized by negative expectations, perceptions about pain and disability, and previous physiotherapy treatment. This is the first study reporting that previous treatment by physiotherapists is a risk factor for long-term sick leave. This has not been reported before and is an interesting finding that deserves more scrutiny.

## Background

Disability and work absence due to low back pain (LBP) present a major public health problem and high economical costs in western societies [1]. The longer a worker is off work with LBP, the lower are their chances of ever returning to work (RTW) [2,3]. Long-term sick leave may reflect degree of disability and complaints; however, being on sick leave may in itself contribute to prolongation of the sick leave. Earlier negative experiences, poor self-judgement of work ability and low self-esteem has been identified as barriers of RTW in disability pensioners [4]. These factors could be strengthened by the absence of work in itself. RTW is therefore not only cost efficient, it may also be therapeutic and crucial for improvement of LBP.

Several interventions have been identified to reduce complaints and disability due to LBP. Even the most efficient methods leave a number of patients without substantial relief, and being unable to RTW [5]. The aim of this study was to identify significant predictors for not being able to RTW after participating in a randomized controlled trial receiving treatments held to represent best medical practice for LBP [6].

The treatment program consisted of Brief Intervention (BI) or BI and a physical exercise program. BI is a light mobilization program where return to normal activity and work is the main goal of the intervention. Previous studies of BI for LBP have shown significant reduction of sick leave compared to treatment as usual [7-11]. Although effective, a substantial proportion of the patients do not RTW. In the European guidelines for the management of chronic LBP, supervised exercise therapy is recommended [5], and addition of exercise to other noninvasive therapies are associated with small improvements in pain and function [12]. A supervised exercise therapy program was therefore added to BI and compared with BI only. No additional effects of the physical exercise program were found on RTW, pain or function [6].

Psychosocial risk factors, including emotional and social problems, disbelief in recovery, and fear-avoidance behaviour, have been shown to predict long-term LBP [13-16]. Treatment success is predicted by several of these factors, the most important of them being subjective ratings of pain intensity and disability, affective parameters, pain related cognitions, health control beliefs, and coping strategies [17-19]. More objective parameters, like medical data and objective work-related factors, appear less important in predicting treatment outcome [17,20]. It appears that subjective evaluations of health status and job satisfaction are more important predictors of RTW than physical aspects of disability and job demands [21]. One of the strongest predictors of RTW seems to be the patients' own belief in RTW. Recovery expectations [22], fear of own abilities to manage work [23], doubts about RTW [24] and intentions of RTW [25,26], are the best determinants of RTW. Expectations and beliefs are also associated with reductions in pain and disability [22-24]. RTW seems to be more dependent on cognitive factors than physical pathology [24,27]. The aim of this study was to identify predictors of non-RTW in LBP patients through a secondary analysis of the randomized controlled trial comparing BI with BI and physical exercise.

## Methods

### Participants

246 patients, 120 men (49%) and 126 women (51%), mean age 41.1 years (SD 10.7), sick listed 8 - 12 weeks for LBP participated in the trial (see table 1 for baseline characteristics).

**Table 1:** Baseline characteristics of all participants (n = 246)

Continuous variables	Mean	SD	Median
Age	41.1	10.7	39
Back pain intensity (1-10)	6.8	2.0	7
Pain during activity (1-9)	5.7	2.1	6
Pain while resting (1-9)	4.1	2.1	4
Roland Morris Questionnaire	8.8	4.3	9

**Categorical variables**	**n**	**%**	

Civil status			
Married/cohabitant	192	78	
Single/widow/divorced	53	21.5	
Education			
Public school 1-12 years	181	73.6	
University/Postgraduate college	41	16.7	
HSCL-25			
Psychological distress (>1.75)	82	33.3	
No psychological distress (<1.75)	151	61.4	
Perceived reduced ability to walk due to complaints			
Inability to walk more than 1 km	88	35.8	
Able to walk more than 1 km	131	53.3	
Sleep problems due to pain:			
Some, often or all the time	193	78.5	
Never or seldom	48	19.5	
Expectations regarding return to work:			
Negative expectations	140	56.9	
Positive expectations	93	37.8	
Physiotherapy prior to participation in the trial:			
Had received PT prior to the trial	141	57.3	
Had not received PT prior to the trial	102	41.5	
Perceived physical workload:			
Constant back strain more than half of the time	183	74.4	
Constant back strain less than half of the time	53	21.5	
Previous sick leave episodes due to LBP:			
One or none previous episodes	107	43.5	
Two or more previous episodes	137	55.7	

### Selection

**Figure 1:**
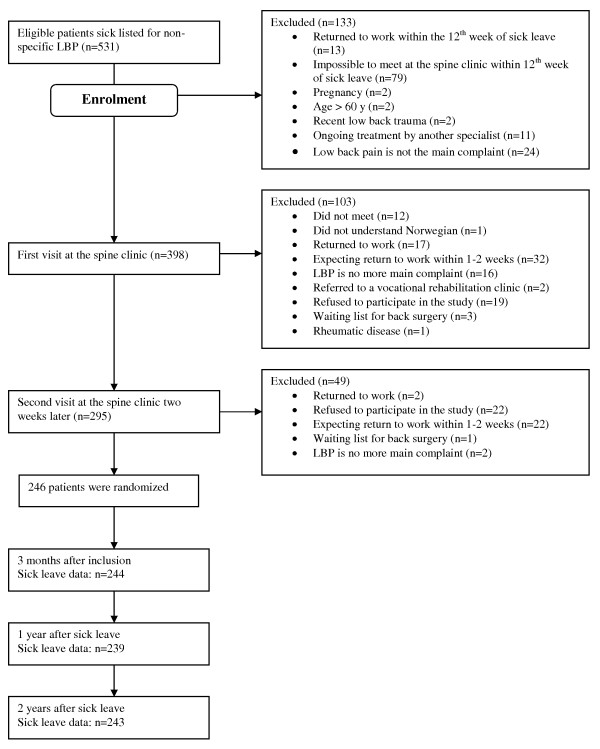
**Flowchart of participation and follow up data**.

During the period from April 2000 to February 2004, The Norwegian Labour and Welfare Administration (NAV) in the 5 neighbour communities of the spine clinic sent written information about the trial and invitation to participate in the study to patients sick listed 8 - 12 weeks, between 18 and 60 years, and diagnosed with one of the following ICPC-diagnoses; L02 (back pain), L03 (low back pain), L84 (back pain without sciatica), and L86 (sciatica). Copies of sickness certificates for 531 participants that had accepted the invitation were then sent to the spine clinic for evaluation for inclusion. 531 patients were referred to the spine clinic and evaluated for inclusion in the study. 133 were excluded before enrolment and 152 were excluded during the first and second visit at the spine clinic. Exclusion criteria were; pregnancy, recent low back trauma, cauda equina symptoms, cancer, osteoporosis, rheumatic low back disease, ongoing treatment for LBP by another specialist, and information from the general practitioner indicating forthcoming RTW (see figure 1). All patients went through a medical examination and filled out questionnaires at baseline and at 3, 12 and 24 months after baseline screening.

### Interventions

BI comprises 2 consultations and a short follow up; first a medical examination by a physician, then a follow up by a physiotherapist immediately afterwards, and finally a brief follow up session with the physician after 2 weeks. For detailed description of the trial see Hagen et al [6]. All included patients received BI at their first visit at the spine clinic. A thorough medical examination was done by the physician (specialist in physical medicine and rehabilitation), and information and feedback given. Unless any serious pathology were found (in which case patients were excluded), patients were informed about the good prognosis and advised to remain active and return to work as soon as possible. The patients were followed up by the physiotherapist and advised and instructed individually on how to train and stretch at home.

At the second visit at the spine clinic, information and advices from the first visit were refreshed, questions answered, and non-excluded patients (n = 246) randomized into two groups, using concealed randomization procedures; BI (n = 122), which received no further treatment, and BI and a physical exercise program (BI/PE) (n = 124). There were no significant differences between the two groups on age, gender or other baseline characteristics. Patients in the BI/PE group participated in an exercise program (one hour, three times a week for eight weeks), designed to "re-educate" the trunk muscle to its normal stabilizing role, improve balance, muscle co-ordination and proprioception [6].

### Questionnaires

All patients answered a number of standard validated Norwegian versions of questionnaires including pain, psychosocial and sociodemographic data. Questionnaires were filled out at the spine clinic at baseline, three months after the second visit, and one and two years after the initial granting of sick leave for both groups. The questionnaires were answered individually by the patients, and were independent of researcher and clinician influence. The following questions and questionnaires were considered to be potential predictors.

Psychological distress was measured by the *Hopkin's Symptom Check list *(HSCL-25) [28], which consists of 25 questions concerning anxiety, depression and somatization. Mean score <1.75 is within the normal range, and a score of 1.75 and above indicate psychological distress in need of treatment. Fear-avoidance beliefs were measured by the *Fear-Avoidance Beliefs Questionnaire(FABQ) *[27]. The FABQ consists of two subscales; fear-avoidance beliefs for physical activity and fear-avoidance beliefs for work. A higher score indicate increased level of fear-avoidance beliefs. Subjective health complaints were measured by 29 items from the *Subjective Health Complaint Inventory *[29]. Subjective somatic and psychological complaints experienced during the last 30 days were measured, with severity scored on a 4-point scale. Self-reported disability was evaluated by the *Roland Morris Questionnaire *[30], where a high score indicates reduced function. An additional question regarding perceived walking distance was also included, asking "How far can you walk before you have to stop due to your complaints?" A revised version of the *Brief Pain Inventory (BPI) *was used to assess pain [31]. Only single items from the BPI were used here. Items regarding pain interference with activities and rest were measured on a scale from 1 (no pain) to 9 (worst possible pain) the last week, while back pain intensity was measured on a scale ranging from 1 (no pain) to 10 (worst possible pain) the last 14 days. Sleep problems due to pain were measured by one question regarding frequency of awakenings during night due to pain. Perceived physical workload was measured by four questions about the frequency work involved repetitive movements, positions with constant strain on the back, hands above shoulder heights, and lifting more than 20 kg. Psychological work load was measured by the *COOPER *job stress questionnaire [32]. The scale consists of 22 items rated on a 6-point scale ranging from 0 (no stress) to 5 (high experience of stress). Coping was measured by the CODE [33]. CODE measures 4 subscales; only instrumental mastery-oriented coping and emotion-focused coping were analyzed here. Treatment prior to participation in the trial was measured by asking the participants about physiotherapy (see table 2 for type of physiotherapy treatment), chiropractic- and fringe medical treatment prior to entering in to the trial. Expectations regarding RTW were measured by asking whether they expected to return to work within the next few weeks or not.

**Table 2:** Type of physiotherapy reported before entering the trial (n = 141)

Physiotherapy treatment	n	%
Local (hot packs, massage, ultrasound etc)	109	77%
Exercise therapy/training	74	52%
Relaxation therapy	26	18%
Home exercise program	31	22%
Other	15	11%

Participants could report more than one option.

### Outcome

Primary outcome was sick leave (due to low back pain) based on register data from local insurance offices and self-report. Non RTW according to register data included patients on sick leave as well as rehabilitation and disability benefit, while non RTW according to the self-reported data was defined by a confirmative response to the question: "Are you sick listed now?". Sick leave status after one and two years were estimated within a timeframe of 14 days before and after the exact date of sick leave. This was done in order to account for patients being put off the sick list just before the date of sick leave, and patients being off the sick list for a few days before a new sick leave period. Data from the insurance offices and self-reported data showed perfect agreement. Information from the local insurance offices was missing on 15 patients, in these cases the self-report data was used to ensure fuller datasets.

### Statistical analyses

SPSS 14.0.2 was used for all the statistical analyses. Since there were no differences between the intervention groups at any follow-ups [6] the groups were merged in all the analyses. RTW/non RTW 3 months after second consultation and 12 and 24 months after initial sick leave were analysed.

All variables were dichotomized using median split. *Expectations of RTW *was divided into "positive expectations" (agreeing to the statement) and "none or negative expectations" (no opinion or disagreeing to the statement), and *sleep problems due to pain *was divided into "seldom or never" and "sometimes, often or all the time". Potential predictors were tested with simple logistic regressions. RTW versus non RTW after 3, 12, and 24 months were the dependent variables (results from the univariate analyses are not reported). All variables with p-values less than 0.01 in the univariate analyses were considered to be potential predictors and were included in the fully adjusted model. Gender, age, education, and treatment group were included as control variables. Stepwise logistic regression was performed to see if the results from the fully adjusted model were consistent. Only patients with complete data were included in the multiple regression models. No imputation methods were therefore applied on any of the single items. However, if subjects had less than 50% missing on a subscale, mean scores on other variables in the subscales were used to compute sumscores.

### Ethical considerations

The study was approved by The Regional Ethical Committee and the Norwegian Social Science Data Services National Register of Data. All principles in the Helsinki declaration were followed. The participants were given declarations of voluntary participation with detailed information. Informed consent was signed by each participant with emphasize on the right to withdraw from the experiment at any time without any explanation.

## Results

No significant differences were found between BI and BI plus exercise on RTW at 3, 12 or 24 months [6], and both intervention groups were therefore merged in the analyses of predictors. Baseline characteristics of all participants in the trial are described in table 1.

No significant differences were found in baseline characteristics or RTW between those included in the multiple regression models (complete datasets) and those excluded from the multiple regression models (not complete datasets).

### Predictors for non-return to work at 3 months

In the adjusted model for 3 months follow up, 72% (n = 176) of the participants had complete data and were included in the analyses. In the fully adjusted model, including possible confounders, pain intensity while resting (odds ratio (OR) = 5.6; 95% CI = 1.7-19.0), perception of constant back strain while working (OR = 4.1; 95% CI = 1.5-11.5), negative expectations of RTW (OR = 4.2; 95% CI = 1.7-10.2), and having been to a physiotherapist (PT) prior to participation in the trial (OR = 3.3; 95% CI = 1.3-8.3) predicted non RTW at 3 months (table 3).

**Table 3:** Regression models of predictors for non-return to work. Adjusted for all the other variables

Adjusted model		3 months (n = 176)		12 months (n = 173)		24 months (n = 175)	
		OR (95%CI)	p- value	OR (95%CI)	p- value	OR (95%CI)	p- value
Group	BI	1.0 (0.4-2.3)	0.96	1.1 (0.6-2.2)	0.77	1.3 (0.7-2.6)	0.45
	BI/PE	1 (ref.)		1 (ref.)		1 (ref.)	
Gender	Male	1.2 (0.5-2.8)	0.75	0.9 (0.4-1.8)	0.74	0.6 (0.3-1.3)	0.20
	Female	1 (ref.)		1 (ref.)		1 (ref.)	
Age	Under 41 years	1.4 (0.6-3.4)	0.42	1.7 (0.8-3.3)	0.16	2.9 (1.4-6.0)	**0.003**
	41 years or older	1 (ref.)		1 (ref.)		1 (ref.)	
Education	Higher education	1.5 (0.5-4.5)	0.49	0.6 (0.2-1.5)	0.27	0.4 (0.2-1.1)	0.08
	No higher education	1 (ref.)		1 (ref.)		1 (ref.)	
Workload	Constant back strain	4.2 (1.5-12)	**0.006**	2.1 (0.8-5.2)	0.11	1.2 (0.5-3.2)	0.65
	Seldom back strain	1 (ref.)		1 (ref.)		1 (ref.)	
Sleep problems	Sleep problems due to pain	1.2 (0.4-3.4)	0.77	1.0 (0.4-2.4)	0.93	1.9 (0.7-5.2)	0.20
	No sleep problems	1 (ref.)		1 (ref.)		1 (ref.)	
Reduced ability to walk	Reduced ability to walk due to pain	2.4 (0.8-7.0)	0.06	2.6 (1.3-5.4)	**0.01**	1.2 (0.6-2.5)	0.63
	Able to walk far	1 (ref.)		1 (ref.)		1 (ref.)	
Expectations of RTW	Negative expectations	4.2 (1.7-10)	**0.001**	1.9 (0.9-4.0)	0.11	2.0 (0.9-4.3)	0.10
	Positive expectations	1 (ref.)		1 (ref.)		1 (ref.)	
Physiotherapy	Had been to PT	3.3 (1.3-8.3)	**0.01**	2.1 (1.1-4.3)	**0.048**	0.8 (0.4-1.7)	0.56
	Had not been to PT	1 (ref.)		1 (ref.)		1 (ref.)	
Back pain intensity (1-10)	> 7 back pain intensity	1.6 (0.5-4.5)	0.41	0.5 (0.2-1.2)	0.13	1.5 (0.7-3.2)	0.32
	< 7 pain intensity	1 (ref.)		1 (ref.)		1 (ref.)	
Pain during activity (1-9)	> 6 pain intensity during activity	1.0 (0.4-2.6)	0.95	2.4 (1.1-5.1)	**0.027**	1.5 (0.7-3.2)	0.32
	< 6 pain intensity	1 (ref.)		1 (ref.)		1 (ref.)	
Pain during rest	> 4 pain intensity during rest	5.6 (1.7-19)	**0.005**	1.8 (0.8-3.8)	0.15	1.5 (0.7-3.3)	0.28
	< 4 pain intensity	1 (ref.)		1 (ref.)		1 (ref.)	

### Predictors for non-return to work at 1-year

In the adjusted model for 1 year follow up, 70% (n = 173) of the participants had complete data and were included in the analyses. In the fully adjusted model, including possible confounders, perceived reduced ability to walk far due to the complaints (OR = 2.6; 95% CI = 1.3-5.4), pain during activities (OR = 2.4; 95% CI = 1.1-5.1), and having been to a PT prior to participation in the trial (OR = 2.1; 95% CI = 1.1-4.3) predicted non RTW at 1 year (table 3).

### Predictors for non-return to work at 2-years

In the adjusted model for 2 year follow up, 71% (n = 175) of the participants had complete data and were included in the analyses. Age below 41 years (OR = 2.9; 95% CI = 1.4-6.0) was the only significant predictor for non RTW after 2 years (table 3).

### Stepwise logistic regression

Backwards stepwise regression was performed in order to test the models with higher power in the analyses. The results confirmed the results as shown in table 3. The same variables predicted non RTW, but with narrower confidence intervals and more consistent findings over time for two of the predictors: Perceived reduced ability to walk far due to the complaints, and the perception of constant back strain while working, were significant predictors at both 3 and 12 months follow up in these analyses.

## Discussion

The aim of this study was to identify predictors of non RTW after Brief Intervention (BI) and BI plus Physical Exercise at a spine clinic. High pain intensity while resting, the perception of constant back strain when working, negative expectations of RTW, and having been to a physiotherapist prior to participation in the trial, were significant predictors of non RTW 3 months after the second visit at the spine clinic. 12 months after initial sick leave, perceived reduced ability to walk far due to the complaints, pain intensity during activities, and physiotherapy prior to participation in the trial, were the strongest predictors for non RTW. Age was the only significant predictor of non RTW 2 years after initial sick leave.

The results are in line with previous studies. Patients' own beliefs regarding RTW [22-26], patients' ratings of pain intensity and disability [17,19,34], and the perception of constant back strain when working [26,35-37], are all well established predictors of non RTW. Gender, age and education did not predict non RTW, except after 2 years where age below 41 years was a predictor. Lower age has been shown to predict non RTW [35], although the opposite finding is more commonly seen [3,21]. Others conclude that the role of sociodemographic parameters remain unclear [17,19].

The surprising finding of this study was that previous treatment by a physiotherapist predicted non RTW. The type of physiotherapy treatment most frequently reported was passive treatments such as hot packs, massage, ultrasound etc (table 2). We have not been able to locate any other studies with similar findings. Although several variables were included in the multiple logistic regression models, there is always the possibility of crucial variables being left out, and therefore not controlled for. Obviously, having been to a physiotherapist could reflect a higher degree of pathology in this group of patients and therefore have nothing to do with the treatment itself. However, in the fully adjusted regression models several variables regarding pain intensity and disability, and variables indicating chronicity like previous sick leave episodes were not found to be significant confounders. The finding could still be a result of selection bias; it is not random who chooses to go to a physiotherapist. These patients may have a more external health locus of control [38], where the power to affect the state of health are believed to be within powerful others or chance, not in the individual. Chance externality, which has been found to predict non RTW in LBP patients [35], could represent expectancies of negative outcomes of actions [39], with the belief that "nothing I do will improve my condition". Externally controlled patients are expected to be more likely to seek treatment. This could therefore explain why previous treatment predicts non RTW. However, why physiotherapy in particular predicts non RTW still remains unanswered. One possibility is that physiotherapy treatment may strengthen unhelpful and pathology promoting beliefs, and may communicate some sort of caution to the patients, warning them about returning to work too soon. A recent qualitative study reports that one of the reasons for calling in sick due to musculoskeletal complaints, was advice given by physiotherapists about staying away from work [40]. Physiotherapists and chiropractors believe more in the myths of LBP than GPs, with patients reflecting corresponding beliefs as their health care provider [41]. Physical therapy for LBP does not always adhere to the evidence-based guidelines, and adherence to the guidelines is associated with better clinical outcomes [42]. The therapeutic alliance between the patient and the physiotherapist before entering into the trial may be stronger than the alliance established through the interventions at the spine clinic, and therapist-patient alliance has been found to be strongly associated with patients' adherence to treatment [43]. Although BI consisted of both a physician and a physiotherapist giving the same message, the patient might still need to decide between two struggling messages regarding RTW. This decision will probably be based on trust and alliance, and may favour the advice given by other physiotherapists before the start of this study. Although plausible, these issues still need to be explored further before any conclusions can be drawn.

A limitation which applies for all prognostic studies is the possibility of omitting important predictors. Variables like work strain, or biological variables like genetics, were not included in this study and can not be ruled out as possible confounders. However, we know that although heavy work has been found to predict duration of sick leave [20], it seems to be the subjective perception more than objective strain in terms of high peak loads, repetitive lifts, or heavy loads that determine the outcome [44]. The results of this study are in line with the notion made earlier about the nature of predictors found in the literature, namely their individual and cognitive characteristics. It is the patients' perceptions about their pain, how far they think they can walk, and whether they expect to be back to work within the next few weeks that determine if they RTW or not.

Many theories have been suggested to explain differences in individual health. The recent Cognitive Activation Theory of Stress (CATS) combines physiological and cognitive explanations [39]. Acquired expectations regarding own abilities to cope, are crucial for health outcomes according to CATS. If the LBP patient has learned that no matter what he or she does, there will be no change in the condition, or all attempts lead to even worse outcomes, the patient has a negative response outcome expectancy (NROE). NROE may cause sustained arousal and thereby negative health consequences, thru for example sensitization [45,46]. Sensitization may be the psychobiological mechanism explaining the individual differences in tolerance and acceptance of common health complaints, explaining why LBP experienced by so many, only endures and disables in a few. The process is understood in terms of neurological and psychobiological sensitization mechanisms, where sustained activation increases the risk of interference with the activity in the pain pathways, contributing to the sensitization [39,45,46]. However, sensitization can also occur in higher forms, as cognitive sensitization. This is believed to cause long-lasting activation and continuing reactivation of specific pain- and illness-related cognitive networks, similar to an attentional bias where too much attention is focused around the pain [47]. A cognitive sensitization of pain-related networks will most likely effect perceptions and beliefs regarding pain and disability, such as the perceptions and beliefs that here was shown to predict non RTW. For the patient this might result in a vicious circle, with negative expectations, sensitization, more pain, lack of coping, and no RTW.

Older patients (above 41 years) were more likely to be back at work at 2 years follow up. Older individuals, still active in the labour market, might have established a positive response outcome expectancy, they have learned that they are successful. They might also have learned to cope with the complaints during the years, making them more robust than the younger patients. Patients with less ability to cope might have disappeared from the labour market at an early age, contributing to a "healthy worker effect" [48], leaving the older cohort better able to cope with work and complaints. Either way; LBP patients' thinking about their complaints seems to be crucial in terms of illness course and RTW. Appropriate treatment for these patients could therefore be cognitive behavioural therapy (CBT), where the aim is to break the vicious circle through directly addressing and challenging the patient's perceptions and beliefs, and to desensitize the pain-related cognitive networks through the change of cognitions and behaviour. The impressive results of BI in terms of RTW [8,49] may be caused by the cognitive approach of the intervention, and corresponds with the positive effects of other interventions where the aim has been to adjust cognitive and behavioural factors [50-52]. Our data correspondingly suggest that a cognitive approach is a reasonable avenue of exploration.

## Conclusion

It appears that return to work is highly dependant on individual and cognitive factors. Patients not returning to work after the interventions were characterized by negative expectations, perceptions about pain and disability, and previous physiotherapy treatment. Physical therapy as predictor of no return to work has not been reported before and is an interesting finding that deserves more scrutiny.

## Competing interests

The authors declare that they have no competing interests.

## Authors' contributions

EMH conceived of the study, and participated in its design and coordination. SER participated in the design of the study, performed the statistical analysis and drafted the manuscript. HRE participated in the design of the study and helped to draft the manuscript. All authors read and approved the final manuscript.

## Pre-publication history

The pre-publication history for this paper can be accessed here:

http://www.biomedcentral.com/1471-2474/10/139/prepub
